# Association Between Dietary Nitrite intake and Glioma Risk: A Systematic Review and Dose-Response Meta-Analysis of Observational Studies

**DOI:** 10.3389/fonc.2022.910476

**Published:** 2022-07-08

**Authors:** Weichunbai Zhang, Jing Jiang, Yongqi He, Xinyi Li, Shuo Yin, Feng Chen, Wenbin Li

**Affiliations:** ^1^ Department of Neuro-Oncology, Cancer Center, Beijing Tiantan Hospital, Capital Medical University, Beijing, China; ^2^ College of Nursing, University of South Florida, Tampa, FL, United States

**Keywords:** nitrite, nitrate, glioma, brain tumor, meta-analysis, dose-response relationship

## Abstract

**Background:**

Nitrite and nitrate intake through food and water may be an important risk factor for many cancers, including glioma. However, the association of nitrite and nitrate with glioma is unclear.

**Objective:**

This review aimed to quantitatively assess the effects of nitrite and nitrate on glioma by meta-analysis.

**Methods:**

A literature search was conducted for available articles published in English using the databases of Embase, Web of Science, PubMed, Medline, and the Cochrane Library up to 24 March 2022. According to heterogeneity, the fixed-effects or random-effects model was selected to obtain the merger’s relative risk (RR). Based on the methods described by Greenland and Longnecker, we explored the dose-response relationship between nitrite/nitrate and the risk of glioma. Subgroup analysis, sensitivity analysis, and publication bias tests were also used.

**Results:**

This study reviewed 17 articles, including 812,107 participants and 4,574 cases. For glioma in adults, compared with the lowest intakes, the highest intakes of nitrite significantly increased the risk of glioma (RR=1.26, 95% confidence interval (95%CI):1.09-1.47). For brain tumors in children, compared with the lowest intakes, the highest intakes of nitrate significantly increased the risk of brain tumors (RR=1.27, 95%CI:1.06-1.52). The results of subgroup and sensitivity analyses remained unchanged. In the dose-response relationship, per 1 mg/day increase in nitrite intake increased the risk of glioma by 14% (RR=1.14, 95%CI:1.01-1.27).

**Conclusions:**

Our analysis suggests that nitrite increases the risk of glioma in adults, while nitrate increases the risk of brain tumors in children. Therefore, the effects of nitrite and nitrate on glioma cannot be ignored.

**Systematic Review Registration:**

https://www.crd.york.ac.uk/prospero/, identifier CRD42022320295.

## Introduction

Compared with other types of cancer, the incidence of brain tumors is low, with an annual incidence of only 22.6/100,000. But primary brain tumors are one of the leading causes of death from cancer, accounting for about 13,000 deaths each year. And one in 1,300 children under the age of 20 is diagnosed with a brain tumor each year (1, 2). The most common pathological subtype of all primary brain tumors is glioma, accounting for approximately 80% of brain tumors (3). However, the current conventional treatment for glioma is not satisfactory and has caused a severe disease burden (4). Therefore, glioma prevention has become a hotspot by identifying and managing related risk factors.

But so far, only ionizing radiation has been considered an established risk factor for glioma (5, 6). For chemical factors, N-nitroso compounds (NOCs) could be found in brain tissue and were one of the most likely contributors to gliomas (7). Nitrite and nitrate in food and water could be metabolized by bacterial flora in the digestive tract to form NOCs, one of the main ways people are exposed to NOCs (8). As people seemed to be unable to avoid nitrates and nitrites ingested orally, there was a growing body of study on nitrites and nitrates and gliomas. Several earlier case-control studies did not find a significant effect of nitrite and nitrate on glioma (9–11). Blowers et al. found that nitrite in cured meat significantly increased the risk of glioma in adults in a Los Angeles community-based case-control study (odds ratio (OR)=2.10, 95% confidence interval (95%CI): 1.00-4.60) (9). However, in a German population study, only NOCs were found to affect glioma (OR=2.80, 95%CI: 1.50-5.30), but nitrite (OR=1.10, 95%CI: 0.60-2.00) and nitrate (OR=0.90, 95%CI: 0.50-1.50) results were not significant (10). Chen et al. also found similar results in the Nebraska Health Study II, for both nitrite (OR=0.80, 95%CI: 0.50-1.30) and nitrate (OR=0.70, 95%CI:0.40-1.20) were not associated with the risk of glioma (11). Lee et al. found that nitrite significantly increased the risk of glioma in men (odds ratio (OR)=2.10, 95%CI: 1.10-3.80), but not in women (OR=1.50, 95%CI: 0.70-3.10) in the hospital-based case-control study (12). Similarly, a significant result for the single-sex population was found in Gile et al.’s study of nitrates and gliomas, but the result was inversely correlated, with nitrates appearing to be protective against gliomas in the female population (OR=0.53, 95%CI: 0.28-0.96) (13). However, in a cohort study of 545,770 participants with an average follow-up of 7.2 years, higher dietary nitrite intake was associated with a 32% increased the risk of glioma (RR=1.32, 95%CI: 1.01-1.71), but the nitrate results were not significant (relative risk (RR)=1.28, 95%CI: 0.97-1.70) (14). In contrast, Barrett et al. analyzed nitrate concentrations in 148 water supply districts in the Yorkshire area of England. They found that regions with higher nitrate concentrations had a higher incidence of brain tumors (RR=1.18, 95% CI:1.08-1.30) (15). In addition, different sources of nitrite and nitrate might also cause differences in outcomes. Mary et al. analyzed the effects of nitrite and nitrate in water and food on glioma and found that only dietary nitrite from plant sources increased the risk of glioma (OR=2.80, 95%CI: 1.00-8.20), with no significant results for other sources (16). A study of nitrite and nitrate exposure during pregnancy leading to brain tumors in children also showed that Pogoda et al. found that nitrite intake through cured meat during pregnancy increased the risk of brain tumors in offspring (OR=3.00, 95% CI: 1.20-7.90) (17). Weng et al.’s study of nitrate exposure in pregnant women’s drinking water found that nitrate also had similar results (OR=1.40, 95%CI: 1.07-1.84) (18). But Bunin et al.’s earlier study in the Children’s Cancer Group registry found that maternal exposure to nitrite and nitrate had no significant effect on neither glioma nor brain tumors in children (13, 19). Due to the rarity of this disease, most studies on the association of nitrite or nitrate with glioma had included a relatively small number of cases, which limited the statistical power of any individual study to detect the association. In addition, nitrite and nitrate were mainly from the environment and diet, and the exposure concentrations of nitrite and nitrate in different studies were not consistent, so the differences in exposure concentrations in a single study might lead to different results, and the previous the meta-analysis did not take into account the dose-response relationship between exposure levels and glioma, which might have contributed to the differences in results between studies.

To quantitatively assess the association of nitrite and nitrate with glioma, we searched all published observational studies on nitrite or nitrate and glioma, using dose-response meta-analyses to quantify the association between nitrite or nitrate intake and the risk of glioma to explore further evidence for the effect of nitrite or nitrate on glioma.

## Methods

### Search Strategy

A literature search was conducted for available articles published in English using the databases of Embase, Web of Science, PubMed, Medline, and the Cochrane Library up to 24 March 2022. The Cochrane Library search terms used for the title, keywords, and abstract were “nitrite” OR “nitrate” OR “N-nitroso compounds” OR “NO_3_” OR “NO_2_” OR “nitrosamine” OR “nitrite amine” combined with “glioma” OR “brain cancer” OR “brain tumor”. Other databases also referred to the same retrieval strategy for literature search. Two investigators searched for relevant articles during the retrieval process and independently reviewed all retrieved studies without document type, language, or other applicable restrictions, and unpublished articles were excluded.

### Inclusion and Exclusion Criteria

For inclusion, studies had to fulfill the following criteria (1): the exposure of interest was nitrite or nitrate intake. These studies provided daily intake of each food or total intake of a food group through food frequency questionnaires or dietary records, and the dietary survey methods were tested for reliability and validity or had been used in previous studies. The individual exposure was calculated based on the unit content of nitrite or nitrate in each food reported in local government reports or published articles; (2) the outcome of interest was glioma in adults; we also considered childish patients, but because most childish patients in the current study had brain tumors as the outcome, the outcome of interest was considered brain tumors when the subjects were children; (3) the study type was observational; (4) the exposed population was adults; pregnant women were exposed in the childhood brain tumor study. Since there were few studies evaluating nitrite and nitrate exposure in children when evaluating the association of nitrite and nitrate with brain tumors in children, the exposed population should be pregnant women, that was, to assess the association between nitrite and nitrate of pregnant women during pregnancy and the risk of brain tumors in offspring.

The exclusion criteria of the meta-analysis were as follows: (1) non-observational study (reviews, case reports, and clinical trials); (2) effect size or 95%CI could not be obtained; (3) animal experiments and cell experiments; (4) if there were multiple studies used the same population, we excluded studies with smaller sample sizes.

### Data Extraction

Two investigators independently assessed eligibility based on inclusion criteria and data extraction. Disagreements were discussed and resolved by a third investigator. For selected studies, data included study characteristics: the first author, year of publication, country, study population (adults or children), study type (cohort study or case-control study), age, disease (glioma or brain tumor), sample size, number of cases, exposure source (food or water), exposure type (nitrite or nitrate), exposure dose, effect size (OR or RR), and 95%CI extracted from the most adjusted model.

### Quality Assessment

The Newcastle-Ottawa Quality Assessment Scale (NOS) was used to assess the quality of the included studies. It was scored independently by two investigators and was discussed by a third investigator in the event of disagreement. Scores ranged from 0 to 9 (20).

### Statistical Analysis

The possible heterogeneity in study results was examined using Cochran Q and quantified by the *I^2^* statistic. In the heterogeneity analysis, *P-values* less than 0.1or *I^2^* greater than 50% were considered to be statistically significant heterogeneity. Pooled estimates based on random-effects models were reported when considerable heterogeneity was detected. Otherwise, pooled estimates based on fixed-effects models were reported. We used these two effect models to calculate pooled RRs and 95% CIs for the highest and lowest nitrate or nitrite intake categories for analysis. In addition, subgroup analyses were performed according to study population (American population and other populations), study design (case-control study and cohort study), study quality (≤7 and >7), and source of exposure (food and water). We used sensitivity analysis to assess each study’s relative impact on the total effect size by successively omitting one study when determining the effect size. To detect publication bias, Egger’s test and Begg’s test were used.

Subsequently, we also explored the dose-response relationship between nitrite/nitrate and the risk of glioma in adults and the dose-response relationship between nitrite/nitrate during pregnancy and the risk of brain tumor in offspring. This study analyzed the dose-response relationship using a method developed by Greenland and Longnecker (21). For this method, we needed to extract at least three groups of the exposure dose of nitrite or nitrate, number of participants, number of cases, effect size, and 95%CI in each study. The corresponding median or mean value of nitrite or nitrate for each group was used for risk assessment for each study. If median or mean values for nitrite or nitrate were not available for each group, in which case the midpoint of the upper and lower limits for each group should be designated as the middle value of the exposure level. If the highest group was open, we should assume the interval width was the same as the second highest group.

All data analyses were carried out by Stata14.0. *P-values* were 2-sided with a significance level of 0.05.

## Results

### Study Characteristics

**Figure 1:**
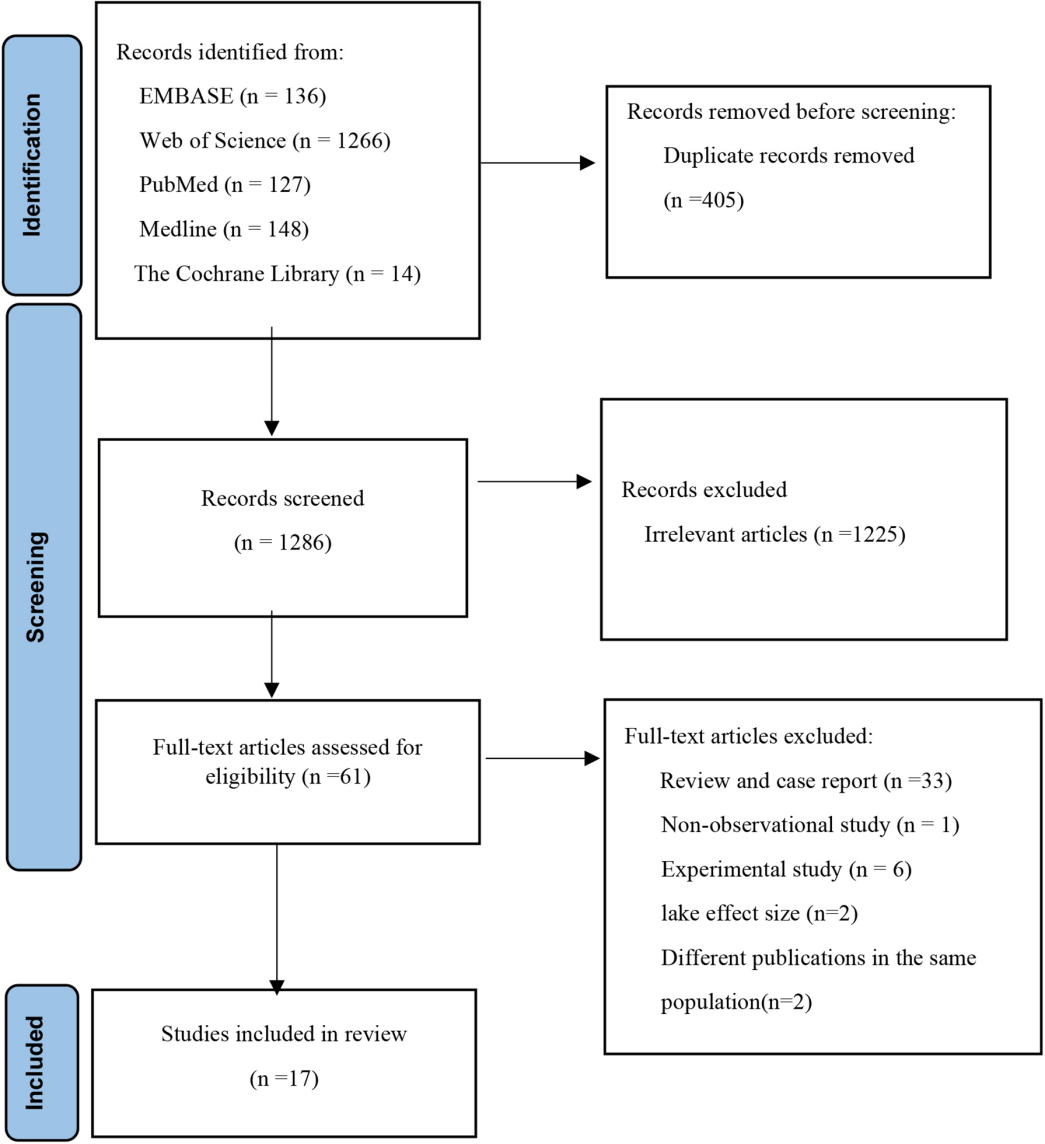
Flow diagram outlining the systematic search and articles selection process.

**Figure 1** shows a flow chart for literature search and study screening. We identified 1691 potentially relevant articles by searching Embase, Web of Science, PubMed, Medline, and the Cochrane Library. After excluding duplicates between different databases, 1286 articles were screened according to title and abstract, and after excluding 1225 articles irrelevant to the study purpose, 61 articles went on full-text review. Of these, 44 articles were excluded due to non-observational studies, experimental studies, lacking effect sizes and 95% CI, and duplication of study populations. Ultimately, 17 articles were included in the meta-analysis.

Characteristics of the 17 included articles are shown in **Table 1** (9–14, 16–19, 22–28). A total of 812,107 participants and 4,574 patients were included in all studies. Most adult participants were between the ages of 18-75, and child participants were between 0-18, and each study included both gender groups. The included studies were mainly concentrated in America, with a few studies completed in Germany, Australia, Israel, China, and Spain, including 15 case-control studies and 2 cohort studies. The primary sources of exposure to nitrite or nitrate were food and water. About 50% of the studies had a NOS score of 8 or higher.

**Table 1:** Characteristics of studies investigating the association between nitrite/nitrate and glioma.

Study	Year	Country	Study type	Age	Sample size	Cases	Disease	Source	Nitrite/Nitrate	Effect size(95%CI)	Quality score
**Adults**											
Boeing (10)	1993	Germany	Case-control	25-75	533	115	Glioma	Food	Nitrite	1.10 (0.60-2.00)	8
									Nitrate	0.90 (0.50-1.50)	
Gile (13)	1994	Australia	Case-control	20-70	818	409	Glioma	Food	Nitrite	1.29 (0.89-1.87)	8
									Nitrate	0.83 (0.57-1.23)	
Blowers (9)	1997	America	Case-control	25-74	188	94	Glioma	Food	Nitrite	1.40 (0.60-3.50)	7
									Nitrate	0.70 (0.20-1.80)	
Lee (12)	1997	America	Case-control	>20	853	432	Glioma	Food	Nitrite	1.83 (1.14-2.95)	8
Schwartzbaum (22)	1999	America	Case-control	44.9	80	40	Glioma	Food	Nitrite	1.60 (0.30-8.00)	7
Chen (11)	2002	America	Case-control	≥21	685	236	Glioma	Food	Nitrite	0.80 (0.50-1.30)	7
									Nitrate	0.70 (0.40-1.20)	
Ward (16)	2005	America	Case-control	≥21	406	121	Glioma	Food	Nitrite	1.10 (0.50-2.50)	7
									Nitrate	1.30 (0.70-2.60)	
Michaud (23)	2009	America	Cohort	25-75	230655	335	Glioma	Food	Nitrite	1.26 (0.89-1.79)	8
									Nitrate	1.02 (0.66-1.58)	
Dubrow (14)	2010	America	Cohort	50-71	545770	585	Glioma	Food	Nitrite	1.32 (1.01-1.71)	7
									Nitrate	1.28 (0.97-1.70)	
**Children**											
Bunin (19)	1993	America	Case-control	0-6	322	166	Brain tumor	Food	Nitrite	1.11 (0.58-2.10)	8
									Nitrate	0.64 (0.34-1.23)	
Lubin (24)	2000	Israel	Case-control	0-18	874	300	Brain tumor	Food	Nitrite	0.97 (0.60-1.70)	9
									Nitrate	1.15 (0.80-1.70)	
Mueller (25)	2001	America	Case-control	0-20	310	119	Brain tumor	Water	Nitrite	8.80 (2.10-46.0)	9
									Nitrate	1.40 (0.10-15.0)	
Pogoda (17)	2001	America	Case-control	0-19	1311	514	Brain tumor	Food	Nitrite	3.00 (1.20-7.90)	7
Mueller (26)	2004	America	Case-control	0-15	820	283	Brain tumor	Water	Nitrite	0.80 (0.40-1.90)	8
									Nitrate	0.80 (0.40-1.50)	
Weng (18)	2011	China	Case-control	0-9	914	457	Brain tumor	Water	Nitrate	1.40 (1.07-1.84)	8
Stayner (27)	2021	America	Case-control	0-15	27186	289	Brain tumor	Water	Nitrate	1.82 (1.09-3.04)	7
Zumel-Marne (28)	2021	Spain	Case-control	10-24	382	79	Brain tumor	Water	Nitrate	1.76 (0.91-3.41)	7

### Effect Size Estimations of the Risk for the Association Between Nitrite/Nitrate and Glioma

**Figure 2:**
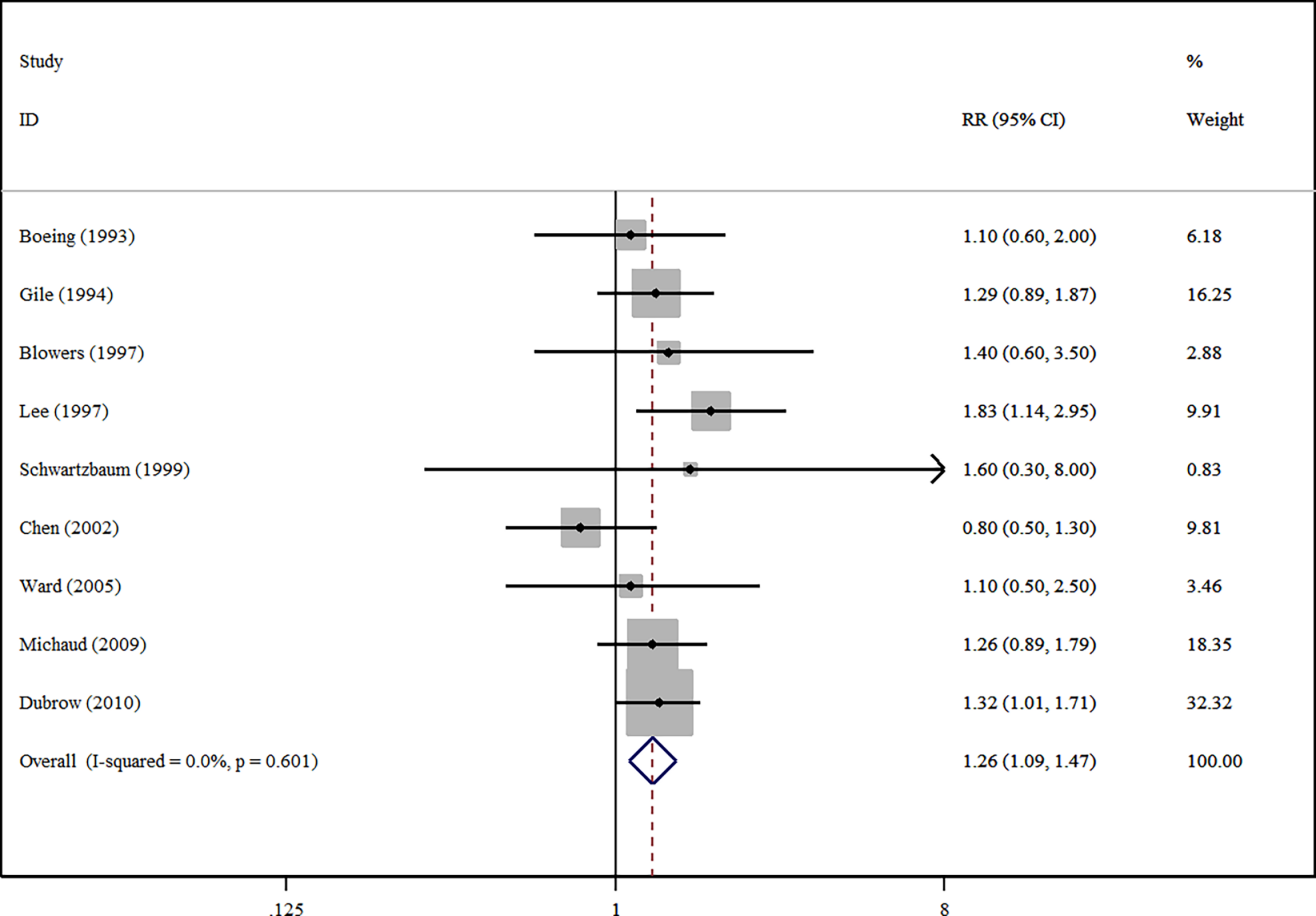
A forest plot showing risk estimates of the association between nitrite and glioma in adults.

**Figure 3:**
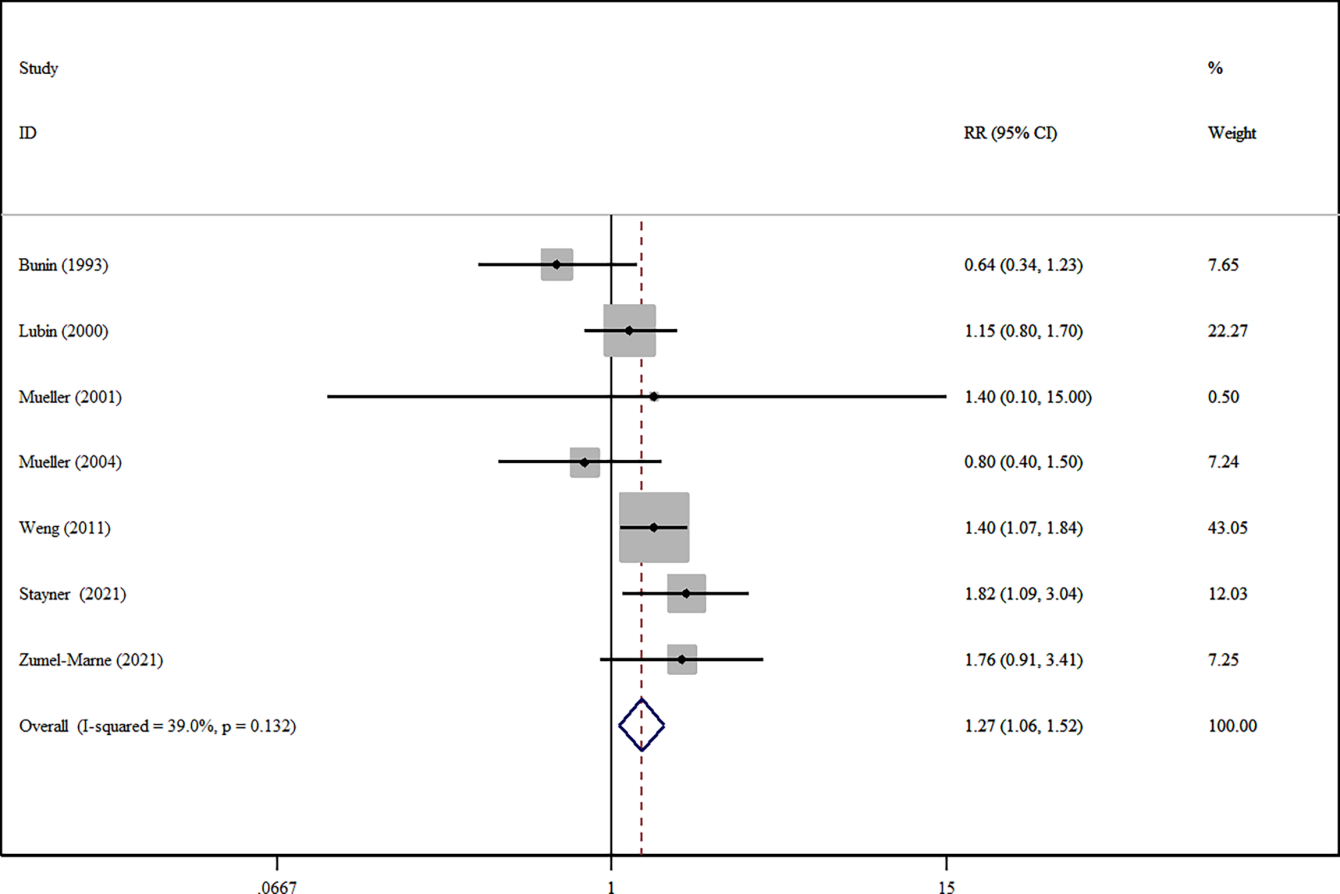
A forest plot showing risk estimates of the association between nitrate and brain tumors in children.

The effect size estimations between nitrite/nitrate and the risk of glioma are shown in **Table 2**. For glioma in adults, compared with the lowest intakes, the highest intakes of nitrite significantly reduced the risk of glioma (RR=1.26, 95%CI:1.09-1.47, *I^2 ^=*0, *P _for heterogeneity_* =0.601) (**Figure 2**). In contrast, the highest intakes of nitrate were not related to the risk of glioma (RR=1.02, 95%CI:0.86-1.21, *I^2 ^=*9.1%, *P _for heterogeneity_* =0.359) (**Supplementary Figure 1**). For brain tumors in children, compared with the lowest intakes, the highest intakes of nitrate significantly reduced the risk of brain tumors (RR=1.27, 95%CI:1.06-1.52, *I^2 ^=*39.0%, *P _for heterogeneity_* =0.132) (**Figure 3**). In contrast, the highest intakes of nitrite were not related to the risk of brain tumors (RR=1.49, 95%CI:0.81-2.74, *I^2 ^=*66.0%, *P _for heterogeneity_* =0.019) (**Supplementary Figure 2**).

**Table 2:** A meta-analysis of the association between nitrite/nitrate and glioma.

	Number of studies	RR (95%CI)	*I^2^* (%)	*P*_for heterogeneity_
Adults				
Nitrite	9	1.26 (1.09-1.47)	0	0.601
Nitrate	7	1.02 (0.86-1.21)	9.1	0.359
Children				
Nitrite	5	1.49 (0.81-2.74)	66.0	0.019
Nitrate	7	1.27 (1.06-1.52)	39.0	0.132

### Subgroup Analysis

For glioma in adults, nitrite was statistically significant in the American population subgroup (RR=1.27, 95%CI:1.07-1.51), the cohort study subgroup (RR=1.30, 95%CI:1.05-1.60), and the study quality score >7 subgroup (RR=1.34, 95%CI:1.09-1.66). Nitrate had no statistical significance in each subgroup, which was consistent with the overall combined results. However, in the study type subgroup, its heterogeneity decreased from 9.1% to 0 (**Table 3**).

**Table 3:** Subgroup analysis for the association between nitrite/nitrate and glioma.

	Subgroup	Number	RR (95%CI)	*I^2^* (%)	*P _for heterogeneity_*
**Adults**					
Nitrite	Study population				
	American population	7	1.27 (1.07-1.51)	3.1	0.402
	Other population	2	1.23 (0.90-1.69)	0	0.659
	Study type				
	Case-control study	7	1.23 (0.99-1.52)	4.0	0.396
	Cohort study	2	1.30 (1.05-1.60)	0	0.835
	Study quality				
	≤7	5	1.19 (0.96-1.47)	0	0.472
	>7	4	1.34 (1.09-1.66)	0	0.527
Nitrate	Study population				
	American population	5	1.10 (0.90-1.35)	15.5	0.315
	Other population	2	0.85 (0.62-1.17)	0	0.813
	Study type				
	Case-control study	5	0.86 (0.67-1.10)	0	0.689
	Cohort study	2	1.20 (0.95-1.52)	0	0.391
	study quality				
	≤7	4	1.13 (0.90-1.41)	34.5	0.205
	>7	3	0.91 (0.70-1.17)	0	0.785
**Children**					
Nitrite	Study population				
	American population	3	2.60 (0.87-7.76)	72.8	0.025
	Other population	2	0.91 (0.59-1.41)	0	0.687
	Study quality				
	≤7	1	3.00 (1.17-7.70)	–	–
	>7	4	1.24 (0.67-2.31)	61.7	0.050
	Source of exposure				
	Food	3	1.33 (0.74-2.36)	53.8	0.115
	Water	2	2.41 (0.23-25.07)	86.5	0.007
Nitrate	Study population				
	American population	3	0.73 (0.46-1.15)	0	0.781
	Other population	4	1.41 (1.16-1.71)	0	0.473
	study quality				
	≤7	2	1.80 (1.20-2.69)	0	0.937
	>7	5	1.17 (0.96-1.43)	37.2	0.173
	Source of exposure				
	Food	2	0.99 (0.72-1.37)	57.9	0.123
	Water	5	1.42 (1.14-1.75)	5.0	0.378

For brain tumors in children, nitrate was statistically significant in the other population (RR=1.41, 95%CI:1.16-1.71), the study quality score ≤7 subgroup (RR=1.80, 95%CI:1.20-2.69), and the water subgroup (RR=1.42, 95%CI:1.14-1.75). And in the subgroup of the study population, the heterogeneity decreased from 39.0% to 0. Nitrite had no statistical significance in each subgroup, which was consistent with the overall combined results (**Table 3**).

### Sensitivity Analysis and Publication Bias

The effect of individual studies on the overall RR was assessed by repeating the meta-analysis after removing each study in turn (**Table 4**). The result was slightly insignificant for nitrates and brain tumors in children when Weng’s study was excluded. For other results, when we removed a single study at a time, none of the studies had an excessive impact on the association between nitrite/nitrate and glioma. This suggested that the results of the meta-analysis were relatively stable.

**Table 4:** Sensitivity analysis and publication bias.

	Influential analysis	Egger’s test	Begg’s test
Nitrite and Glioma in adults	1.03-1.56	0.859	1.000
Nitrate and Glioma in adults	0.72-1.30	0.225	0.649
Nitrite and Brain tumor in children	0.67-4.26	0.067	0.221
Nitrate and Brain tumor in children	0.93-1.62	0.609	0.764

Publication bias was assessed by Egger’s test and Begg’s test. All publication bias *P-values* were >0.1, indicating that the difference was not statistically significant, so the publication bias of this study was not significant (**Table 4**).

### Dose-Response Relationship

**Figure 4:**
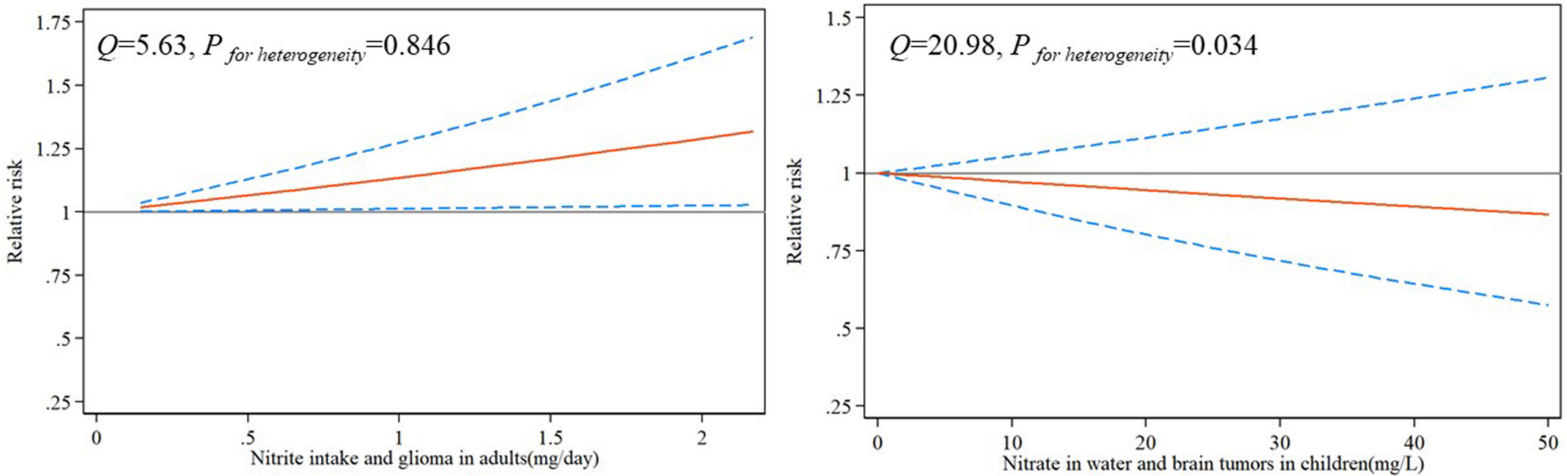
Risk between nitrite/nitrate and glioma estimates from dose-response meta-analysis.

Due to the limited number of available articles, 7 articles could only analyse dose-response relationships. The dose-response relationship between nitrite intake and the risk of glioma in adults was shown in **Figure 4**. There was a significant linear dose-response relationship between nitrite and glioma, with per 1 mg/day increase in nitrite intake increasing the risk of glioma by 14% (*P_-nonlinearity_* =0.999, RR=1.14, 95%CI:1.01-1.27). However, the results were not significant for the dose-response relationship between nitrate and brain tumors in children (*P_-nonlinearity_* =0.392, RR=0.97, 95%CI:0.90-1.06).

## Discussion

Based on 17 observational studies on nitrite/nitrate and gliomas published from 1993 to 2021, involving 812,107 participants and 4,574 cases, our meta-analysis results suggested that nitrite could significantly increase the risk of glioma in adults, while nitrate could significantly increase the risk of brain tumors in children. However, nitrate had no significant effect on gliomas in adults, and nitrite had no significant effect on brain tumors in children. For the results of the dose-response relationship, per 1 mg/day increase in nitrite intake was associated with a 14% increased risk of glioma.

We performed subgroup analyses based on the study population, study type, study quality, and source of exposure to explore sources of heterogeneity. For gliomas in adults, nitrite heterogeneity was 0, suggesting that pooled results were robust and significant in most subgroups. The heterogeneity of nitrates might be mainly due to differences in study types. Five of the seven studies were case-control studies, and dietary survey methods specifically assessed nitrate intake. These case-control studies used the food frequency questionnaire method was used to conduct dietary surveys, which may cause some heterogeneity due to recall bias. For brain tumors in children, differences in the study population may be a source of heterogeneity in nitrates. In the subgroup of the study population, the heterogeneity decreased from 39.0% to 0. Since most nitrates in the study came from drinking water, it might be due to the different concentrations of nitrate in water in different regions. For nitrite, heterogeneity decreased slightly when Pogoda’s study was excluded, which may be related to the relatively low quality of the study. Sensitivity analysis showed that most of the study results were stable. No publication bias was found in all studies.

Our study found that a higher nitrite intake was associated with a significantly increased risk of glioma in adults (RR=1.26, 95%CI:1.09-1.47), whereas nitrate did not have similar results (RR=1.02, 95% CI: 0.86-1.21). This was not the same as the results of the previous meta-analysis. In this study, the relationship between nitrite (OR=1.17, 95%CI:0.98-1.37, *I^2^ =*0) and nitrate (OR=1.11, 95%CI:0.91-1.31, *I^2^ =*0) and glioma was not significant, but the number of this study was small, and only three articles were included. Therefore, this might not contradict our results (29). The association between dietary nitrite intake and the risk of glioma had been controversial in many previous studies, with most case-control studies (9–11), showing that nitrite was not associated with the risk of glioma (12). A case-control study based on the American population found that nitrites significantly increased the risk of glioma in adults (OR=2.10, 95%CI: 1.00-4.60), and these nitrites were mainly from cured meats (9), Another European study also did not find the effects of nitrite (OR=1.10, 95%CI: 0.60-2.00) and nitrate (OR=0.90, 95%CI: 0.50-1.50) on glioma, but higher intake of NOCs was found to increase the risk of glioma by 180% (OR=2.80, 95%CI: 1.50-5.30) (10). However, Lee et al. found that higher nitrite intake in men with lower vitamin C intake was still a risk factor for glioma (OR=2.10, 95%CI:1.10-3.80) (12). And the results of a prospective cohort study by Dubrow et al. showed that high intake of nitrite was a risk factor for adult glioma (RR=1.32, 95%CI: 1.01-1.71), but nitrate had no such effect (RR=1.28, 95%CI: 0.97-1.70) (14). Although another cohort study did not find significant results for the development of nitrite on gliomas (RR=1.26, 95% CI: 0.89-1.79) (23), this was not contradictory. The two studies differed in the source of the content for dietary nitrite. The content for dietary nitrite in the study by Dubrow et al. was obtained by reviewing the literature focusing on food in America and Canada, while the content for dietary nitrite in the study by Michaud et al. was derived from European literature, which may vary in assessing dietary nitrite intake in the American population. Although nitrite and nitrate formed NOCs in the body and increased cancer risk, there were still some differences in the metabolic pathways. Compared with nitrite, nitrate needed to be reduced to nitrite by bacterial flora in the oral cavity and alimentary canal before further forming NOCs in the alimentary canal (30). Therefore, the time required for nitrate to form NOCs *in vivo* was significantly longer than that of nitrite. In healthy people, ingested nitrates were usually excreted rapidly in urine, but nitrites were barely detectable in urine, suggesting that nitrites were more easily passed out of the body (31–33). So, this might lead to the formation of more NOCs from nitrite in the body than from nitrate. This might be one of the main reasons for the difference in results between nitrite and nitrate. Furthermore, the insignificant results for nitrate might be due to the protective effect of vegetables in the diet. According to the results of the population dietary survey, nitrite in food mainly came from processed animal food (about 40%) (14, 34), while nitrate mainly came from vegetables (90%) (14). Vegetables contained nutrients that inhibit N-nitrosation in the body, such as antioxidants, including vitamin C and vitamin E. Experiments had shown that vitamin C could inhibit the formation of N-nitroso compounds from nitrates in human subjects (35). Therefore, although a large amount of nitrate was ingested through vegetables, the concurrent intake of vitamin C could block nitrosation by quenching free radicals, thereby inhibiting tumorigenesis (36).

The incidence of brain tumors in children is about 6.06/100,000 (37). Therefore, most studies on the risk factors of childhood brain tumors did not use a single pathological subtype as the disease outcome (18, 27, 28). However, glioma is still the most common pathological subtype of brain tumors in children (38). Our study found that maternal nitrate intake during pregnancy significantly increased the risk of brain tumors in children (RR=1.27, 95%CI: 1.06-1.52). This was consistent with some previous findings. Stayner et al. through the Danish National Cancer Registry found that maternal exposure to higher concentrations of nitrate before pregnancy was associated with an increased risk of brain tumors in children (OR=1.82, 95%CI: 1.09-3.04). Still, the exposures in both prenatal (OR=1.65, 95%CI:0.97-2.81) and postnatal (OR=1.48, 95%CI:0.82-2.68) stages were not significant in a nested case-control study (27). By comparing nitrate concentrations in drinking water sources, Ouattara et al. found that children in areas with 2.1-5 mg/L had a higher incidence of brain tumors than regions with nitrate concentrations of 0-2 mg/L (39). Zumel-Marne et al. analyzed the lifetime cumulative residential nitrate of 364 participants from three countries and found that nitrite intake of 41.70-97.00 mg/day significantly increased the risk of brain tumors in children (OR=2.12, 95%CI:1.02-4.40), but the result of higher intake was not significant (OR=1.72, 95%CI:0.82-3.63) (28). However, Lubin et al. analyzed the association between dietary nitrate exposure of Israeli pregnant women and brain tumors in offspring and found no significant results (OR=1.15, 95%CI: 0.80-1.70) (24). Similarly, Bunin et al. did not find the effect of nitrate in the diet on brain tumors in the study of the Children Cancer Group (OR=0.64, 95%CI:0.34-1.23) (19). Although a meta-analysis of seven studies also found that higher nitrate concentrations in water were associated with a greater risk of brain tumors (RR=1.15, 95% CI:1.06-1.24), the study included both adults and children; no single population analysis was performed (40). Only two previous studies had shown that higher maternal nitrite intake during pregnancy was associated with an increased risk of brain tumors in children (17, 25). Still, our results showed no effect of nitrite on brain tumors in children (RR=1.49, 95%CI: 0.81-2.74). From this finding, the effects of nitrite/nitrate on brain tumors in children and gliomas in adults appeared to be the opposite. The possible reasons were mainly as follows. On the one hand, because there was a small proportion of non-glioma patients in childhood brain tumors, nitrite did not seem to influence other brain tumors. Mueller et al. found that nitrite exposure had a significant effect on astrocytoma in children (OR=5.70, 95%CI: 1.20-27.20), but not on primitive neuroectodermal tumors (OR=1.30, 95%CI: 0.10-30.90) and other brain tumors (OR=0.70, 95%CI: 0.10-7.70) (26). Similar results were found in other studies (19). On the other hand, most studies on brain tumors in children were based on maternal exposure during pregnancy (17, 26, 27). There were differences in the metabolism of nitrite and nitrate in the general population and pregnant women. Animal experiments had shown that oral nitrite in pregnancy rats, whose offspring were almost free of brain tumors (41). And the source of nitrate in this part of the study was mainly drinking water, not vegetables, lacking the protective effect of vegetable intake, thereby reducing the reverse effect on nitrate.

Our study was the largest meta-analysis to explore the association between nitrite/nitrate and glioma. This study had some advantages. First, based on the articles published so far, we explored for the first time the dose-response relationship between nitrite and glioma risk. We found that an increase in nitrite intake per 1 mg/day was associated with a 14% increase in the risk of glioma. Second, we explored the exposure of nitrite/nitrate in adult and pediatric populations and found differences in the effects of nitrite/nitrate on the two populations, providing new ideas for future glioma prevention. Moreover, the heterogeneity of our significant results was less than 40%, demonstrating that the results were relatively reliable. However, the study still had several limitations. We found that, due to the low incidence of brain tumors in children, most studies on nitrite/nitrate in the pediatric population used brain tumors as disease outcomes rather than a specific pathological subtype. This may lead to slightly different results in the adult population. But more than half of the brain tumors in children were still gliomas. And the exposure routes were relatively consistent, all of these were maternal exposure during pregnancy. So, the results still had some reference value. Similarly, limited by the number of cases of adult glioma subtypes (glioblastoma, astrocytoma, etc.), analysis of the effects of nitrite and nitrate on glioma subtypes was not possible. Secondly, nitrite and nitrate exposure were quantified mainly through dietary surveys, which could not avoid bias caused by recall. Most studies on the determination of nitrite and nitrate content in food and water were based on government testing or literature reports, moreover, most of the studies only evaluated the exposure of a certain pathway, which seemed to be unable to represent the overall exposure level of the population. In the future, the association of biomarkers of nitrate and nitrite exposure *in vivo* with glioma should also be explored, to reduce the recall bias caused by the dietary surveys. Thirdly, since nitrite and nitrate from plant foods, meat, and water appeared to have different effects on gliomas, future studies should also further explore the relationship between different sources of nitrite and nitrate and glioma. Furthermore, these studies were mainly case-control studies, which cannot verify the time of association, but our study had included in the most relevant literature published.

## Conclusion

The current meta-analysis suggests that nitrite increases the risk of glioma in adults, and nitrate increases the risk of brain tumors in children. Therefore, the effect of nitrite/nitrate on glioma cannot be ignored. In future studies, we should find a method to evaluate nitrite/nitrate exposure accurately and further explore the relationship between nitrite/nitrate and glioma.

## Data Availability Statement

The original contributions presented in the study are included in the article/**Supplementary Material**. Further inquiries can be directed to the corresponding authors.

## Author Contributions

WL and WZ contributed to the conception or design of the work. WZ, JJ, and YH contributed to searching the databases. WZ, JJ, and XL contributed to the acquisition, analysis, or interpretation of data for the work. WZ, XL, and SY proofread and modified the language. WL and FC reviewed and edited the manuscript. All authors contributed to the article and approved the submitted version.

## Funding

This study was supported by the Special Research Project of Capital Health Development 2016 (No. 2020-2-2048) and National Science and Technology Major Project of China (No. 2016ZX09101017).

## Conflict of Interest

The authors declare that the research was conducted in the absence of any commercial or financial relationships that could be construed as a potential conflict of interest.

## Publisher’s Note

All claims expressed in this article are solely those of the authors and do not necessarily represent those of their affiliated organizations, or those of the publisher, the editors and the reviewers. Any product that may be evaluated in this article, or claim that may be made by its manufacturer, is not guaranteed or endorsed by the publisher.
