# Supplementary material for: Association Between Dietary Nitrite intake and Glioma Risk: A Systematic Review and Dose-Response Meta-Analysis of Observational Studies
Source: Front Oncol. 2022 Jul 8;12:910476. doi: 10.3389/fonc.2022.910476 (PMC9304866; doi:10.3389/fonc.2022.910476)
Supplement: Supplementary file 1 [file DataSheet_1.docx]

Supplementary Material


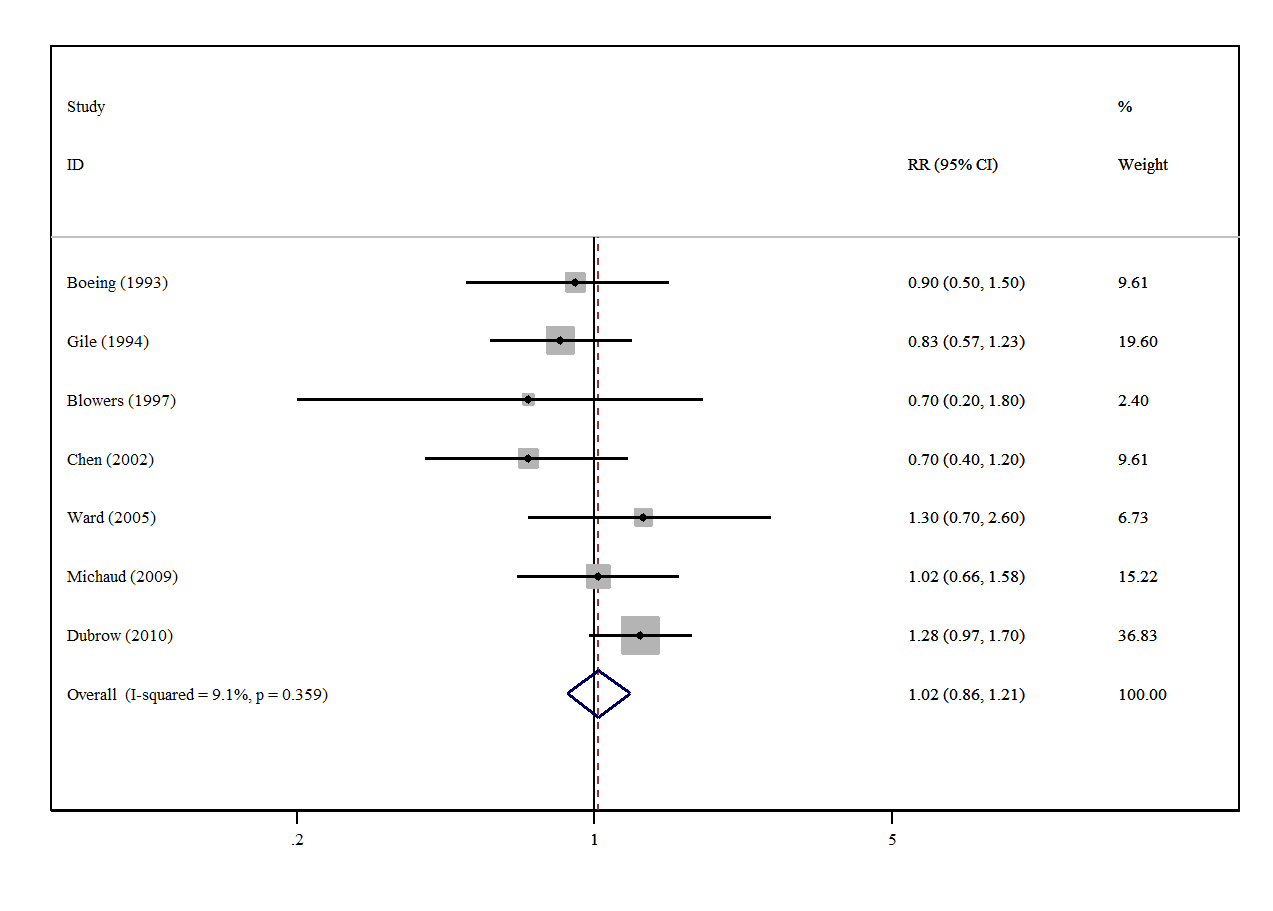
**Supplementary Figure 1 |** A forest plot showing risk estimates of the association between nitrate and glioma in adults.


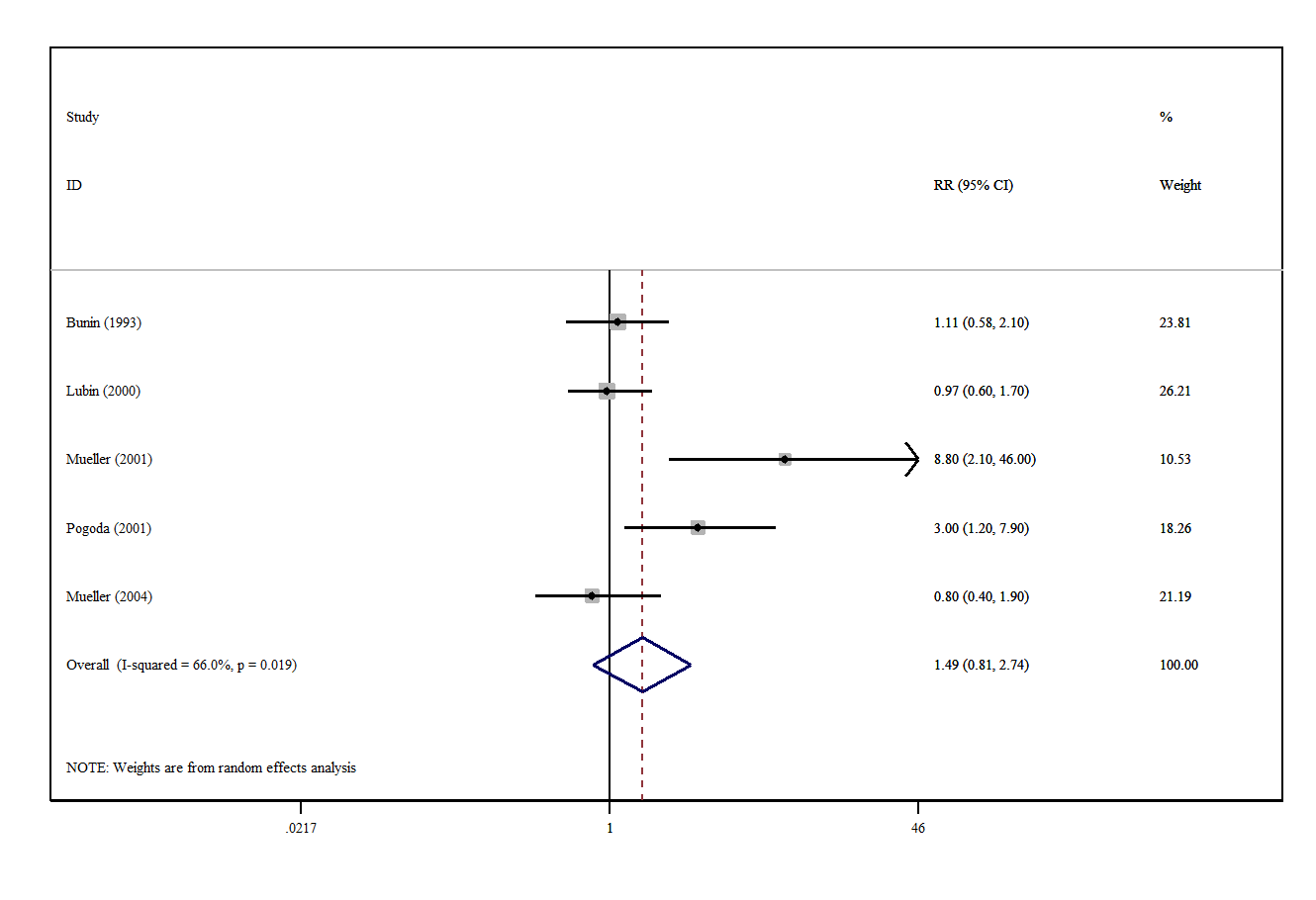
**Supplementary Figure 2 |** A forest plot showing risk estimates of the association between nitrite and brain tumors in children.

**Supplementary Table 1|** The main results and publication bias in different statistical models.

|  | Fixed-effects model | Random-effects model | Egger’s test | Begg’s test |
| --- | --- | --- | --- | --- |
| Nitrite and Glioma in adults | 1.26(1.09-1.47) | 1.26(1.09-1.47) | 0.859 | 1.000 |
| Nitrate and Glioma in adults | 1.02(0.86-1.21) | 1.01(0.84-1.21) | 0.225 | 0.649 |
| Nitrite and Brain tumor in children | 1.23(0.89-1.71) | 1.49(0.81-2.74) | 0.067 | 0.221 |
| Nitrate and Brain tumor in children | 1.27(1.06-1.52) | 1.23(0.95-1.59) | 0.609 | 0.764 |
